# Thymidine phosphorylase expression in normal, hyperplastic and neoplastic prostates: correlation with tumour associated macrophages, infiltrating lymphocytes, and angiogenesis

**DOI:** 10.1038/sj.bjc.6600281

**Published:** 2002-05-06

**Authors:** E Sivridis, A Giatromanolaki, I Papadopoulos, K C Gatter, A L Harris, M I Koukourakis

**Affiliations:** Department of Pathology, Democritus University of Thrace, Alexandroupolis, Greece; Department of Urology, Democritus University of Thrace, Alexandroupolis, Greece; Nuffield Department of Clinical Laboratory Sciences, John Radcliffe Hospital, Oxford OX3 7LJ, UK; Molecular Oncology Laboratories, Institute of Molecular Medicine, John Radcliffe Hospital, Oxford OX3 9DS, UK; Department of Radiotherapy/Oncology, Democritus University of Thrace, Alexandroupolis, Greece

**Keywords:** thymidine phosphorylase, tumour-associated macrophages, infiltrating lymphocytes, angiogenesis, prostate specific antigen, prostate carcinoma

## Abstract

Thymidine phosphorylase is an angiogenic factor primarily expressed by cancer cells, stromal cells and tumour-associated macrophages in many human malignancies. These different types of thymidine phosphorylase-expressing cells, however, may have a distinct place in the angiogenic process, and this question was addressed in the present study. A series of 20 normal/hyperplastic prostate glands and 60 prostate carcinomas was investigated by immunohistochemistry, using specific antibodies for thymidine phosphorylase (P-GF.44C), tumour-associated macrophages (CD68), endothelium (CD31) and prostate specific antigen (ER-PR8). Thymidine phosphorylase expression by normal and hyperplastic epithelial or stromal cells occurred almost exclusively in the context of an intense lymphocytic infiltrate. High thymidine phosphorylase cancer cells and thymidine phosphorylase stromal cells expression was associated with high angiogenesis in prostate carcinomas, and this significant association was extended to include both tumour-associated macrophages and tumour-infiltrating lymphocytes. Thymidine phosphorylase expression and tumour-infiltrating lymphocytes were related inversely with prostate specific antigen reactivity. In conclusion, thymidine phosphorylase is a major angiogenic factor in prostate carcinomas and its up-regulation is likely to occur in the context of a host immune response.

*British Journal of Cancer* (2002) **86**, 1465–1471. DOI: 10.1038/sj/bjc/6600281 www.bjcancer.com

© 2002 Cancer Research UK

## 

Angiogenesis is the formation of new capillaries from a pre-existing vascular network (Folkman, 1985). It occurs as a physiological phenomenon in regeneration and repair, and as a pathological process in the genesis and progression of cancer. Paracrine stimuli, such as thymidine phosphorylase (TP) and vascular endothelial growth factor (VEGF), released from cancer cells, stromal cells and activated macrophages play an important role in the regulation of tumour angiogenesis. Once activated by these stimuli, endothelial cells proliferate, migrate and, eventually, differentiate into functioning capillaries (Pinedo and Slamon, 2000). In prostate carcinomas, as in other tumours, an increased angiogenesis, assessed as microvessel density (MVD), is usually connected with poor prognosis (Hall *et al*, 1994; Bettencourt *et al*, 1998; Borre *et al*, 1998; Offersen *et al*, 1998; Strohmeyer *et al*, 2000a,b).

Thymidine phosphorylase is an important angiogenic stimulus in prostate carcinomas and, as would be expected, it is associated with high MVD (Sugamoto *et al*, 1999; Okada *et al*, 2001). The molecule, which is also known as platelet-derived endothelial cell growth factor (PD-ECGF), catalyses the reversible phosphorylation of thymidine to thymine and 2-deoxy-D-ribose-1-phosphate (Fox *et al*, 1995b; Griffiths and Statford, 1997). The mechanism of the angiogenic activity is through generation of free radicals by the metabolic product 2-deoxy-D-ribose which was shown to stimulate endothelial cell migration and new blood vessel formation (Moghaddam *et al*, 1997; Brown and Bicknell, 1998; Brown *et al*, 2000). The free radicals activate expression of VEGF, interleukin-8 (IL8) and matrix metalloproteinase-1 (MMP1) (Brown *et al*, 2000).

The macrophages are highly differentiated cells of the mononuclear macrophage system which, after activation, assume a variety of specific functions, including phagocytosis (Adams and Hamilton, 1984; Elsbach and Weiss, 1988; Klebanoff, 1988), antigen presentation (Auger and Ross, 1992) chemotaxis (Brown and Gallin, 1988) and release of important secretory products. In malignant disease, the process of macrophage activation was connected with an anti-tumour activity (Lavnikova *et al*, 1996; Ragnhammar, 1996) and stimulation of angiogenesis (Polverini and Leibovich, 1984; Arras *et al*, 1998), the latter being mediated through the secretion of extracellular matrix degrading enzymes and several angiogenic cytokines, including the TP/PD-ECGF (Sunderkotter *et al*, 1994; Leek *et al*, 2000). Tumour associated macrophages (TAMs) express TP, but recruitment of activated macrophages into the tumour by tumour cells expressing TP may also contribute to angiogenesis. A chemotactic response by monocytes/macrophages to TP has been reported (Miyazono and Takaku, 1991).

The present study was undertaken in order to investigate the expression, distribution and the role played by the different types of TP-expressing cells, including TAMs and tumour-infiltrating lymphocytes (TILs), in stimulating angiogenesis in prostate adenocarcinomas. In addition, TP expression was analysed for possible associations with histological grade and PSA expression.

## MATERIALS AND METHODS

Twenty biopsy and autopsy specimens from normal and hyperplastic prostate glands, and sixty surgical specimens of prostate adenocarcinoma were retrieved from the files of the Department of Pathology, Democritus University of Thrace, Greece. The tissues had been fixed in 10% formol saline and processed through graded alcohols to paraffin wax. Histological diagnosis of prostate cancer was based on haematoxylin and eosin stained sections. The Gleason system (Gleason, 1966) was used for histological grading. A primary and secondary Gleason score (1 to 5) was determined for every tumour, and the combined score (Gleason sum) was then calculated. To obtain sufficient quantities for statistical analysis, the tumours were grouped in three categories: low grade (well differentiated) if the combined Gleason score was 4 or less; intermediate grade (moderately differentiated) if combined Gleason score were 5, 6 or 7 (3+4, with a majority of Gleason 3 areas and a small proportion – less than 20% – of Gleason 4 components); and high grade (poorly differentiated) if the Gleason sum was 7 (4+3) or above.

### Immunohistochemistry

Immunohistochemistry was performed at 3 μm thick formalin-fixed, paraffin-embedded sections with monoclonal antibodies to: angiogenic factor TP, pan-endothelial antigen CD31, TAMs, and prostate specific antigen (PSA). Details are given in Table 1

**Table 1:**
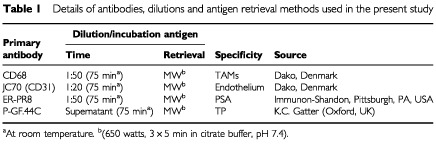
Details of antibodies, dilutions and antigen retrieval methods used in the present study

. A standard streptavidin-biotin immunoperoxidase method was used. Endogenous peroxidase activity was blocked by exposure to absolute methanol containing 3% hydrogen peroxide. Antigen retrieval was achieved by microwave heating. Non-specific background staining was reduced by protein blocking agent. The primary antibodies were applied for 75 min at room temperature. The sections were then sequentially incubated with: (a) anti-rabbit anti-mouse antibody (Kwik Biotinylated Secondary, Immunon, Shandon, Pittsburgh, PA, USA) for 15 min, and (b) Kwik Streptavidin peroxidase reagent (Immunon, Shandon, Pittsburgh, PA, USA) for 15 min. Antibody reactivity was detected with 3,3′-diaminobenzidine (DAB) as chromogen. The slides were counterstained with Mayer's haematoxylin. Known positive controls were included in each staining run. Omission of the primary antibody and replacement by normal rabbit serum at the same concentration was used as negative controls.

### Assessment of TP and TAMs

TP expression was assessed in epithelial cells (normal, hyperplastic and neoplastic), stromal cells and in tumour-associated macrophages. The percentage of TP positive epithelial cells, i.e., epithelial cells with strong cytoplasmic and/or nuclear reactivity, was recorded for each case. The median value was used to define cases into groups of high and low TPcc reactivity.

The expression of TP by stromal fibroblasts and smooth muscle cells (TPsc) was recorded in all optical fields. The percentage of optical fields with strong TPsc expression was recorded, and the median value was used to define groups of high and low TPsc reactivity.

The number of tumour-associated macrophages (TAMs) was determined at ×400 magnification. Counting was performed in three tumour areas of high macrophage concentration (hot spot areas). The mean was calculated. Areas of necrosis were excluded from TAMs quantification.

### Assessment of lymphocytic infiltration

The extent of stromal lymphocytic infiltration was assessed on haematoxylin and eosin stained sections. Areas of necrosis were excluded from this evaluation. The percentage of optical fields (×200) with a prominent lymphocytic response in the tumour stroma was recorded, forming the lymphocyte infiltration index (LII). The median value was used to define groups of high and low LII.

### Assessment of angiogenesis

Angiogenesis was assessed by microvessel counting in three tumour areas of high vascular density (hot spot areas) at ×200 magnification. Only vessels with a well defined lumen or a linear vessel shape were taken into account. The mean of these vessel counts obtained was the microvessel density (MVD). Using the median MVD as a cut-off point, our cases were grouped into categories of low and high MVD.

### Statistical analysis

Statistical analysis and graphic presentation were performed using the GraphPad Prism 2.01 package (GraphPad, San Diego, CA, USA, www.graphpad.com). The Fisher's exact test or the unpaired two-tailed *t*-test was used for testing relationships between categorical variables as appropriate. A *P*-value <0.05 was considered significant.

## RESULTS

### Expression of TP in normal and hyperplastic prostates

**Figure 1:**
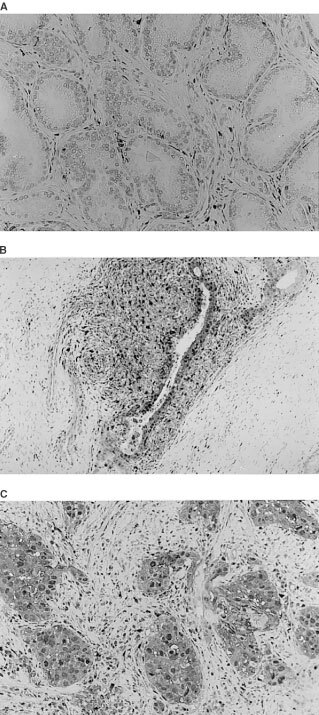
(**A**) Normal prostate gland, free of lymphocytic infiltrates, lacking TP expression from ducts, acini and stroma. (**B**) Normal prostatic acini, densely infiltrared by lymphocytes, exhibiting nuclear and cytoplasmic TP expression by ‘epithelial’ (basal and secretory) and stromal cells. (**C**) Prostate carcinoma showing a strong nuclear/cytoplasmic reactivity by tumour and tumour supporting stromal cells.

Normal and hyperplastic prostatic glands were, by and large, unreactive to P-GF.44C (Figure 1A

). However, in areas of intense lymphocytic infiltration both glandular and stromal cell component expressed TP. The expression was strong and mixed nuclear/cytoplasmic (Figure 1B). A similarly strong nuclear/cytoplasmic reactivity was invariably expressed by the normal, inflammatory cell free, epithelium of the adjacent seminal vesicles.

### Expression of TP in prostate carcinomas

TP was primarily expressed by cancer cells (TPcc), stromal cells (TPsc), of both fibroblastic and smooth muscle origin, endothelial cells (TPec) and in tumour associated macrophages (TAMs). Lymphocytes were also expressing TP but the extent of lymphocytic positivity could not be confidently assessed in the presence of an intense stromal TP reactivity. In all cases, the pattern of TP expression was mixed nuclear/cytoplasmic (Figure 1C).

The percentage TPcc ranged from 0 to 90%, with a median of 12.5%. Using this value as a cut off point, 30 cases were of low and 30 cases of high TPcc reactivity.

TPsc reactivity ranged from 0 to 100% of the optical fields examined. The median value was 20%. A high TPsc expression (>20%) was noted in 30 of the 60 cases, and was associated with the invariable presence of TP positive TAMs (30 out of 30 cases) and the frequent expression of TPec (12 out of 30 cases *vs* 0 out of 30 cases with low TPsc reactivity) (*P*<0.0001).

### Macrophage and lymphocytic infiltration

The number of CD68 positive cells enumerated in each case ranged from 5 to 55 per ×400 optical field. The median macrophage number (macrophage index, MΦI) was 11, and the mean value±s.d. was 19±10. Using the median value, 30 cancer cases were of high and 30 of low MΦI. The staining reaction for CD68 was distinctive granular and cytoplasmic.

Lymphocytic infiltration of the tumour supporting stroma was recorded in 0–100% (median 25%) of the ×200 optical fields analysed per specimen (assessment of the whole specimen). The median value was used to define the groups of high (24 cases) and low (36 cases) lymphocyte infiltration index (LII).

### Angiogenic correlations

The median microvessel density (MVD) was 22 (range 7–69). Using this value cases were divided into groups of low (30 patients) and high MVD (30 patients). The staining reaction of endothelial cells was mixed membranous/cytoplasmic.

A high TP reactivity by cancer and endothelial cells was significantly associated with increased angiogenesis in prostate tumours (TPcc *P*=0.02; TPec *P*=0.002) (Table 2

**Table 2:**
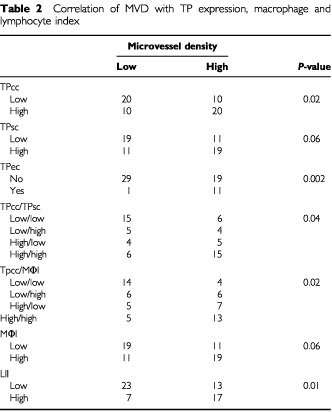
Correlation of MVD with TP expression, macrophage and lymphocyte index

). High LII was also linked with high MVD (*P*=0.01). The association between TPsc expression and MVD, and between the number of TAMs and MVD was marginal (TPsc *P*=0.06; TAMs *P*=0.06).

Using double stratification for TPsc/TPcc, and TPsc/MΦI a significant angiogenic co-operation between TPsc and TPcc (*P*=0.04) was shown, and also between TPsc and TAMs (*P*=0.02) (Table 2). Using continuous variable analysis, tumours with high MVD had a significantly higher mean MΦI than those having low MVD (mean MPI 22±12 *vs* 16±8; *P*=0.02).

### MΦI and TP expression

There was a significant relationship between TPcc and TPsc expression (*P*=0.004; Table 3

**Table 3:**
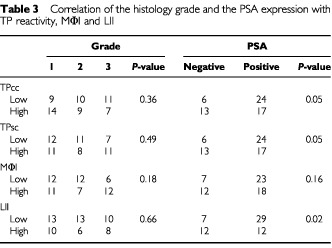
Correlation of the histology grade and the PSA expression with TP reactivity, MΦI and LII

). Although prostate carcinomas with high MΦI were frequently accompanied with high TPcc and TPsc reactivity, this difference did not reach the level of statistical significance (*P*=0.19). Using the MΦI as a continuous variable, a high MΦI was significantly related with TPsc reactivity (22±11 *vs* 16±9; *P*=0.04), but this was not true in the case of TPcc although there was a similar tendency (21±11 *vs* 17±9; *P*=0.10). Further, a strong association between high LII and high MΦI was noted (*P*<0.0001). A high LII was also related with high TP expression by cancer (*P*=0.06) and stromal cells (*P*=0.01).

### Correlation with histological grade and PSA expression

Out of the 60 cases of prostate cancer, 19 (32%) did not show any reactivity after staining for PSA and were considered as negative. The remaining showed a varying degree of strong cytoplasmic reactivity (range 25–100% of cells) and these cases were considered as PSA positive.

Neither TPcc nor TPsc expression was associated with histological grade in prostate carcinomas, but both TPcc and TPsc expression was inversely associated with the expression of PSA (*P*=0.05) (Table 4

**Table 4:**
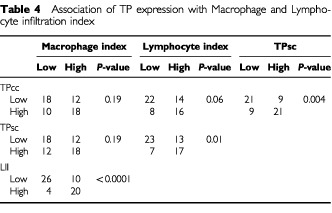
Association of TP expression with Macrophage and Lymphocyte infiltration index

). A significant inverse association between LII and PSA expression was also noted (*P*=0.02). MΦI was more frequent in prostate tumours with high histological grade and loss of PSA, but none of these differences was of statistical significance.

## DISCUSSION

Thymidine phosphorylase/platelet-derived endothelial cell growth factor (TP/PD-ECGF) is a potent angiogenic molecule stimulating endothelial cell migration and new blood vessel formation (Moghadam *et al*, 1995). The enzyme's angiogenic activity has been consistently shown in a variety of human malignancies, despite variations of its principal source of production. Thus, expression of TP by cancer cells was shown in carcinomas of the breast (Fox *et al*, 1995a; Toi *et al*, 1995), lung (Koukourakis *et al*, 1997), head and neck (Giatromanolaki *et al*, 1998) and stomach (Giatromanolaki *et al*, 2000), whereas a predominant stromal cell TP expression was shown in colorectal (Takebayashi *et al*, 1996) and endometrial carcinomas (Sivridis *et al*, 1999), particularly at the invading tumour front. In all cases, however, high TP expression was associated with high angiogenesis, irrespective of the main source of TP production, and this was confirmed by further studies in endometrial and in non-small cell lung tumours, where cancer cells and stromal fibroblasts were independently associated with high angiogenesis (Koukourakis *et al*, 1998; Sivridis *et al*, 2001).

Despite our general understanding, the role of TP in prostate cancer remains inadequately studied. Sugamoto *et al* (1999) indicated that TP levels are higher in prostate carcinomas than in normal prostatic tissues. Okada *et al* (2001) found that TP is expressed in stromal cells, but not in cancer cells, and that an intense stromal TP reactivity in these tumours was associated with high microvessel density. Okada's immunohistochemical study was performed using two monoclonal antibodies (MoAb), the HCT116 and the P-GF-44C.

The present study was based on the MoAb P-GF-44C. TP was detected extensively (up to 90% of cells) in the nuclei and the cytoplasm of cancer cells in half of the 60 cases examined. The intensity of staining was strong, and there was a direct association between high TP expression and high angiogenesis in prostate carcinomas. This observation contrasts the results reported by Okada' group (2001). P-GF-44C is a reliable and very sensitive antibody and such a discrepancy is difficult to be explained. Equally difficult is the explanation required for Okuda's finding that normal prostates express TP in 80% of the cases examined. In our series, normal/hyperplastic prostate glands and stroma were persistently unreactive to P-GF-44C, and a strong nuclear/cytoplasmic TP expression became only apparent in areas of intense lymphocytic infiltration, probably in the context of a chronic prostatitis. Lymphocytes produce a variety of lymphokines, such as interferons and interleukins, which are potent stimulators of TP expression (Schwartz *et al*, 1992; Eda *et al*, 1993; Goto *et al*, 2001). Certainly, this would explain our finding that extensive lymphocytic infiltration of the prostatic tumour stroma is related with strong TP expression by cancer and stromal cells. The pathway by which lymphocytes, or specific subpopulations of lymphocytes, trigger the expression of TP is not clear, although there are reports implicating CD4 positive cells in tumour angiogenesis (Blotnick *et al*, 1994) and natural killer (NK) cells in complex interactions with angiogenic factors (Melder *et al*, 1996). Furthermore, a direct association between lymphocytic and macrophage stromal infiltration was noted. These observations provide strong evidence that up-regulation of TP in the normal, hyperplastic and neoplastic prostate tissues occur in the context of a local host immune response against inflammatory or tumour antigens.

It should be noted in this respect that an intense TP reactivity was consistently detected in normal seminal vesicles, regardless of the presence or otherwise of an inflammatory cell response. The vesicle epithelium, however, is endowed with abundance of mitochondria, a rich rough endoplasmic reticulum and a prominent Golgi apparatus suitable for a vigorous synthetic activity (Nistal *et al*, 1992). Fructose, in particular, is excessively secreted by the vesicle epithelium (Kise *et al*, 2000). Catalytic TP-mediated transformation of thymidine to thymine results in 2-deoxyribose-1-phosphate production (Brown *et al*, 2000). Given that ribose is actively involved in fructose metabolism, a regulatory role for TP may be anticipated in this metabolical function (Ozeki *et al*, 1999; Sawada *et al*, 2000).

It is of interest, however, that despite the general disparity between our results and those of Okada, this group of investigators did find that TP expression by stromal cells is directly related to increased microvessel density. Furthermore, we found that simultaneous expression of TP by cancer and stromal cells was considerably enhanced the angiogenic activity. This synergistic angiogenic effect of the two cellular elements is probably due to the higher intratumoral TP levels achieved in these cases. Nevertheless, the oxidative stress induced by 2-deoxyribose-1-phosphate promotes secretion of VEGF and interleukin-8 angiogenic molecules (Brown *et al*, 2000). Both proteins are angiogenic for prostate cancer (Strohmeyer *et al*, 2000b; Doll *et al*, 2001; Kim *et al*, 2001) and may act synergistically.

Another important observation of the present study is the clear association between the expression of TP and the accumulation of CD68 positive macrophages (TAMs) in the tumour supporting stroma. This finding, which is in accordance with a previous report of ours on non-small cell lung carcinomas (Koukourakis *et al*, 1998), reinforces the view for a chemotactic activity of TP on mononuclear cells (Miyazono and Takaku, 1991). TP-mediated macrophage chemotaxia may, therefore, contribute to the angiogenic process, as activated macrophages, apart from producing TP, usually secrete a variety of pro-angiogenic and angiogenic cytokines (Sunderkotter *et al*, 1994; Ono *et al*, 1999; White *et al*, 2001). The angiogenic relevance of an intense macrophage infiltration of the tumour stroma has been shown in a variety of other human tumours including breast (Leek *et al*, 2000), endometrial (Hashimoto *et al*, 2000) and lung carcinomas (Takanami *et al*, 1999), gliomas (Nishie *et al*, 1999) and malignant melanomas (Torisu *et al*, 2000).

Nevertheless, the role of macrophages in the angiogenic process is far from clear, as an angio-suppressive effect for TAMs has also been established. Metalloelastase of macrophage origin, has been reported as essential for the production of ‘angiostatin’, a potent endogenous suppressor of angiogenesis (Dong *et al*, 1997) and an inhibitor of tumour progression, at least in colorectal carcinomas (Yang *et al*, 2001). Furthermore, an intense infiltration of TP-expressing macrophages in the tumour stroma was related to an improved survival in patients with colorectal tumours (Saito *et al*, 2000). Production of the angiogenic inhibitor thrombospondin 1 by angiogenic macrophages further underlines the anti-angiogenic potential of macrophages (Di-Pietro and Polverini, 1993).

This dual angiogenic/angiosuppressor potential of macrophages emphasises the necessity of identifying specific subpopulations of TAMs, which may have distinct roles in the angiogenic process. The recently reported strong association of high macrophage index with a better post-operative outcome in prostate cancer patients contrasts the postulated ‘angiogenic role’ of macrophages (Shimura *et al*, 2000). High angiogenesis is an important variable of poor prognosis in prostate carcinomas, as has been convincingly shown in a large number of studies (Silberman *et al*, 1997; Bettencourt *et al*, 1998; Borre *et al*, 1998; Strohmeyer *et al*, 2000; Mehta *et al*, 2001). Specific intratumoural conditions (cytokines released by cancer cells, fibroblasts or even by tumour related lymphocytes) may form a complex code that switches on/off or even reverses the macrophage angiogenic machinery.

Finally, the present study revealed a significant inverse relationship between PSA expression and the expression of the angiogenic factor TP in cancer and stromal cells. Although the loss of PSA expression is rather suggestive of cellular de-differentiation, neither TP nor PSA was related to Gleason's score. This lack of association between TP and histological grade is also a feature of other malignancies, including non small cell lung carcinomas and squamous cell carcinomas of the head and neck (Giatromanolaki *et al*, 1997, 1998). In a previous study, we reported that PSA was associated with low microvessel density in prostate carcinomas (Papadopoulos *et al*, 2001). This suppressive effect of PSA on angiogenesis is probably accomplished by a mechanism of converting Lys-plasminogen to biologically active angiostatin-like fragments with an action similar to angiostatin (Heidtmann *et al*, 1999). However, reduced levels of TP in prostate carcinoma expressing PSA suggests that additional hypotheses are needed in order to explain the direct association of PSA with poor vascularity. Whether suppression of the PSA-gene leads directly to an activation of the angiogenic cascade or whether PSA suppression simply co-exists with the up- or down-regulation of angiogenesis controlling genes requires further investigation.

In conclusion, TP is a potent angiogenic factor stimulating new blood vessel formation in prostate carcinomas. Tumour angiogenesis is, primarily, induced by angiogenic stimuli released by neoplastic cells, fibroblasts and smooth muscle cells but, in addition, TAMs and infiltrating lymphocytes may be actively involved in the angiogenic process, at least in a subset of carcinomas. Thymidine phosphorylase may be a target for cytotoxic and anti-angiogenic therapeutic strategies in prostate tumours.
